# Comparative Evaluation of Various Implant Overdenture Attachment Systems in Terms of Survival Rate, Tissue Response, and Patient Satisfaction: A Systematic Review and Meta-Analysis

**DOI:** 10.7759/cureus.83838

**Published:** 2025-05-10

**Authors:** Shailee Patel, Kalpesh Vaishnav

**Affiliations:** 1 Department of Prosthodontics, Crown, and Bridge, Karnavati School of Dentistry, Gandhinagar, IND

**Keywords:** implant-supported overdentures, overdenture attachments, overdentures, survival rate, tissue response and patient satisfaction

## Abstract

Edentulism is a common issue among the elderly, and implant-supported overdentures have been shown to enhance retention, stability, and overall patient satisfaction. The selection of an appropriate attachment system plays a crucial role, as different attachments can impact peri-implant tissue conditions, survival rates, and clinical outcomes. This systematic review and meta-analysis aimed to compare various attachment systems for mandibular implant overdentures based on tissue response, survival rate, and patient satisfaction. A comprehensive electronic and manual literature search was conducted using PubMed, Cochrane Central, and ScienceDirect databases. The review included randomized controlled trials and crossover clinical trials published between 2010 and 2020, with a minimum follow-up period of one year. The risk of bias was evaluated using the Cochrane Collaboration's tool, and statistical analysis was performed using RevMan 5.4 (The Cochrane Collaboration, London, UK). A total of 14 studies met the inclusion criteria, all exhibiting a low risk of bias. Overall, bar attachments demonstrated superior retention, while telescopic attachments were associated with the highest levels of patient satisfaction. Locator attachments proved advantageous for cases with limited interarch space and angulated implants. In contrast, magnetic attachments exhibited the least retention and were associated with more soft tissue changes. The analysis did not reveal a significant correlation between implant survival rates and the type of attachment used.

## Introduction and background

Edentulism remains a prevalent issue among the elderly, often leading to challenges in complete denture adaptation due to inadequate denture-supporting tissues, ridge resorption, and decreased salivary flow [1,2]. Overdentures have emerged as a viable solution, eliminating the instability of conventional dentures by utilizing remaining natural teeth, roots, or implants for enhanced retention, stability, and support [2].

Implant-supported overdentures (ISODs) have significantly improved prosthodontic outcomes, offering better functional efficiency and aesthetics compared to conventional dentures [3]. The use of attachment systems in ISODs further enhances retention and stability, ultimately influencing patient satisfaction and implant longevity [4]. These attachment systems vary in design, mechanics, and clinical performance, affecting peri-implant tissue response, plaque accumulation, gingival health, and marginal bone loss [5,6].

Despite their advantages, ISODs are associated with challenges, including implant positioning, prosthetic design, and attachment selection [4]. The choice of an attachment system is crucial as it affects long-term prosthetic success, maintenance requirements, and patient comfort [4]. However, due to continuous advancements and commercial variations in attachment designs, clinicians often face difficulty selecting the most appropriate attachment for specific cases [7].

This systematic review and meta-analysis aims to evaluate the clinical efficacy of different implant overdenture attachment systems from 1991 to 2021 on the basis of patient satisfaction, tissue response, and survival rates. The findings will assist clinicians in making evidence-based decisions for optimal attachment selection tailored to individual patient needs.

Overdenture attachments enhance implant-supported prosthesis retention, stability, and support [2,8,9]. Bar and telescopic systems offer superior stability, while stud and magnetic attachments suit minimal ridge resorption [4,10,11]. Stud attachments, like ball and locator systems, are widely used for their ease of maintenance and retention [9,12]. Magnetic attachments allow free movement but provide the least retention. Telescopic attachments offer excellent frictional retention but require precise fabrication [10,11]. Attachment selection depends on bone availability, inter-arch space, patient needs, and cost, ensuring optimal treatment outcomes.​

## Review

Methods

This study was carried out according to the recommended reporting items for the systematic review and meta-analysis standards.

Study Protocol

**Figure 1:**
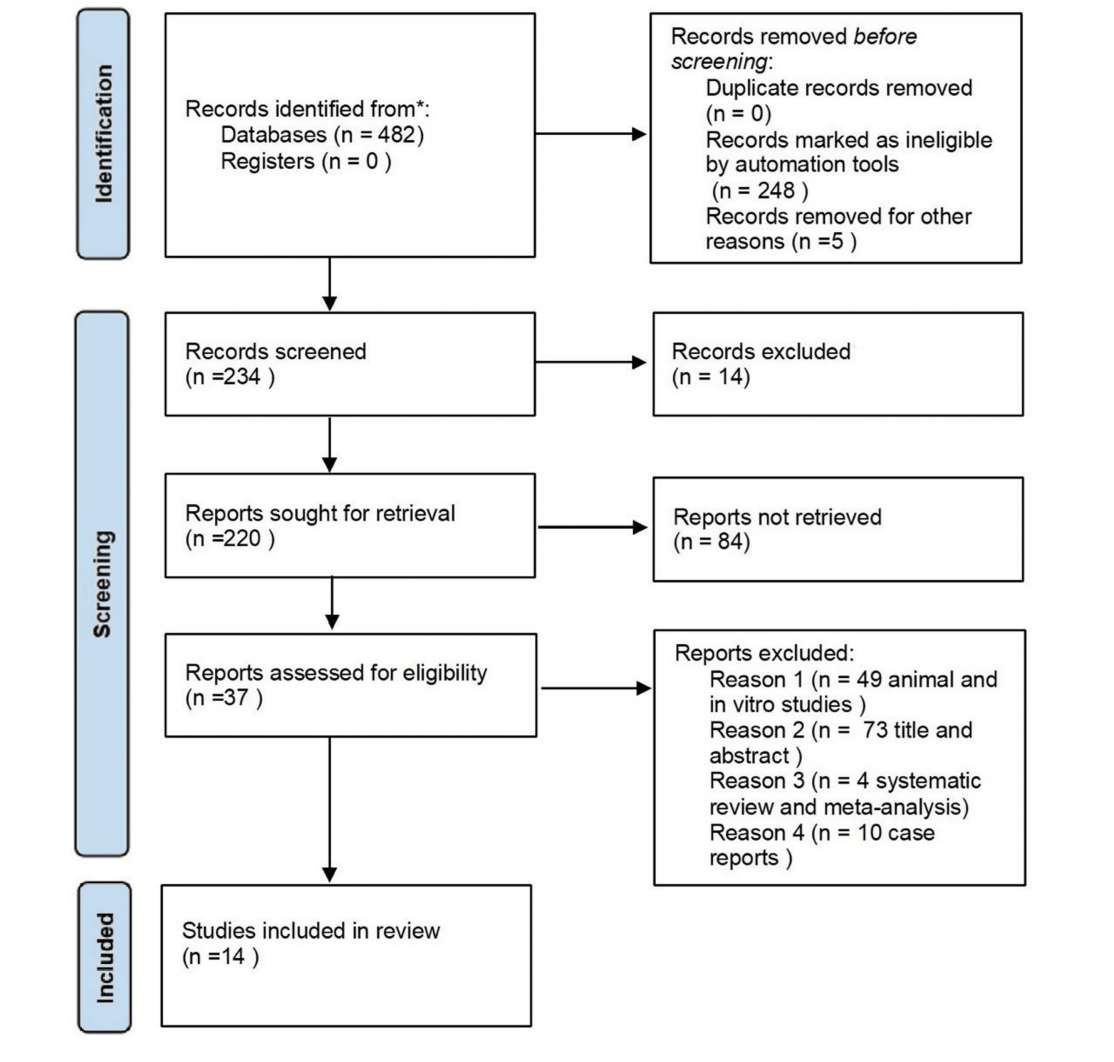
PRISMA flowchart of the included studies PRISMA: (Preferred Reporting Items for Systematic Reviews and Meta-Analyses)

The Centre for Evidence-Based Medicine offered the PICOTS format, which was created as part of a systematic literature search to address the study issue. PICOTS denotes the following: P (patient or population), with the patient having a completely edentulous mandibular arch; I (intervention), with implant-supported or implant-retained overdenture with a minimum of two implants in the mandibular arch; C (comparison), with various attachments for implant-supported/retained overdentures, including stud, bar, magnet, and telescopic; O (outcome) comprising the (1) survival rate of implant, (2) tissue response, and (3) patient satisfaction; T (time), with clinical studies published between 1991 and 2021 and with a follow-up period of a minimum of one year; and S (study design), with in vivo studies involving the mandibular arch, including comparative prospective and randomized clinical trials. Figure 1 depicts the PICOTS format according to the PRISMA (Preferred Reporting Items for Systematic Reviews and Meta-Analyses) 2020 guidelines.

Study Design

Comparative prospective case-control studies, crossover clinical trials, and randomized clinical trials (in vivo studies) involving mandibular implant-supported/retained overdentures with a minimum follow-up of one year were included.

Search Strategy

Three electronic databases were searched: MEDLINE (via PubMed), Google Scholar, and Cochrane Library for studies published between 1991 and 2021 (last search: January 18, 2022). The search used MeSH terms with Boolean operators (‘AND’, ‘OR’, ‘NOT’), focusing on overdentures, denture attachments, and implant-supported dentures.

Two independent investigators screened 482 studies, first filtering by year (1991-2021) and language (English only), excluding 248 studies. After full-text screening, 199 human studies remained, with four systematic reviews/meta-analyses excluded. Further eligibility assessment resulted in 37 full-text articles, from which 23 were excluded based on inclusion/exclusion criteria. A total of 14 studies were included in the final review.

Additionally, reference lists were checked, and hand-searching was conducted in key journals (1991-2021), including the Journal of Prosthetic Dentistry, International Journal of Prosthodontics, and Journal of Oral Implantology. A manual search in Google Scholar confirmed the selection of the study. Finally, all included studies underwent validity assessment and data extraction.

Study Selection and Intervention

The inclusion criteria for this study encompass research published between 1991 and 2021, with studies available only in the English language and conducted on human subjects. The focus is strictly on the mandibular arch, specifically implant-retained overdentures supported by a minimum of two implants. Additionally, the overdenture attachments must be placed on conventional endosseous root-form implants. Eligible studies should discuss the survival rates of various attachments and implants with a minimum follow-up period of one year. Furthermore, studies that examine the comparison of tissue response around attachments and patient satisfaction concerning different attachment-retained implant overdentures are included.

The exclusion criteria eliminated studies involving a partially edentulous arch with implant-supported prostheses and those focusing on maxillary overdenture implant attachments. Studies investigating single-implant-retained overdentures or overdenture attachments placed on short implants are also excluded. Additionally, systematic reviews, meta-analyses, case reports, case series, in vitro studies, and animal studies do not qualify. Studies with a follow-up period of less than one year or those published in languages other than English are also excluded from consideration.

Data Extraction and Collection

The full-text articles were evaluated carefully, and data were extracted and recorded. According to the following criteria, the following data were recorded: name of author, publication year, type of study design, number of patients, average number of implants per patient, follow-up period, type of attachment used in overdenture, outcome parameter, and risk of bias assessment.

All included studies underwent risk of bias assessment by two independent investigators using the Cochrane Handbook guidelines. The Cochrane Collaboration's tool categorized studies as low, high, or unclear risk across seven domains: random sequence generation, allocation concealment, blinding (participants, personnel, and outcome assessors), incomplete outcome data, selective reporting, and other biases. Bias was assessed based on study methods, with randomization, blinding, and allocation concealment determining the risk level.

Statistical Analysis

Statistical tests were conducted using Review Manager software (RevMan 5.4, The Cochrane Collaboration, London, UK). Continuous data were reported as mean difference (MD) with a 95% confidence interval (CI). Heterogeneity was evaluated using Chi², I², and Tau² statistics, with I² > 25% [13], p < 0.05 in Chi², or Tau² > 0 indicating heterogeneity.

Results

The studies identified through electronic data search (PubMed, Google Scholar, and Cochrane Library) using the following MeSH terms and Boolean operators ‘AND’ ‘OR’‘NOT’: “Overdenture ‘AND’ Attachment denture” ‘OR’ “Overdentures ‘AND’ Denture attachment” ‘OR’ “Implant supported denture ‘AND’ Attachment denture”, “mandibular overdenture ‘NOT’ maxillary overdenture ‘NOT’ single implant denture.” The total number of studies identified from the keyword search was 482. The studies done from 1991 to 2021 were included, so 248 studies were excluded. After the initial search, a total of 234 studies were screened, and 49 studies were excluded as they were done on animals and in vitro studies. The total number of studies done on humans was 199. Another 73 studies were excluded after screening the title and abstract, and four systematic reviews and meta-analyses were excluded. This study did not include case reports, so another 10 studies were excluded. After the final search, a total of 37 full-text studies were accessed to determine eligibility. A total of 23 articles were excluded depending on the inclusion and exclusion criteria. Finally, 14 studies were included based on the inclusion criteria (Table 1).

**Table 1:** Characteristics of the included studies The table shows a concise explanation of the study design, number of patients, average number of patients, average implants per patient, follow-up, type of attachment used, and outcome parameters. ITI: International Team for Implantology; TG: trans-gingival; 2ISB: 2 implant-supported bar attachments; 4ISB: 4 implant-supported bar attachments; BEGO: Bremer Goldschlägerei GmbH & Co. KG; CM LOC: Cendres+Métaux Locator

No.	Author	Year	Study design	No. of patients	Avg. Implant per patient	Follow-up	Type of attachment	Outcome parameters
1.	Naert et al. [14]	1998	Prospective randomized controlled clinical trial	36	2 (Branemark System, Nobel Biocare)	1, 4, 6, 12, 24, 36, 48, 60 months	Dolder bar, magnet, ball (Dala Bona)	Tissue response, patient satisfaction, retention
2.	Naert et al. [14]	1999						
3.	Wismeijer et al. [15]	1999	Randomized controlled clinical trial	110	2-4 (ITI dental implant system, Straumann)	3, 9, 19 months	Ball, single bar, triple bar	Tissue response
4.	Gotfredsen and Holmet [16]	2000	Prospective randomized controlled clinical trial	26	2 (Astra Tech dental implant)	1, 2, 3, 4, 5 years	Bar, ball	Tissue response, survival rate
5.	Assad et al. [17]	2004	Randomized controlled clinical trial	10	2 (Dyna implants)	6, 12, 18 months	Bar clip, magnet	Tissue response
6.	Kleis et al. [18]	2009	Prospective case-control	60	2 (Osseotite TG Standard implants)	1 year	Self-aligning Locator, ball	Tissue response, patient satisfaction
7.	Cristache et al. [19]	2009	Randomized controlled trial	46	2 (Straumann implants)	5 years	Ball, magnetic attachment	Tissue response
8.	Burns et al. [20]	2011	Prospective randomized controlled trial	30	2, 4 (standard implants)	1 year	2ISB, 4ISD, ball	Tissue response, patient satisfaction, retention
9.	Krennmair et al. [21]	2012	Prospective randomized controlled trial	51	2 (Camlog, Germany)	1, 2, 3, 12, 24, 36 months	Telescopic crown, milled bar	Tissue response, survival rate
10.	Geckili et al. [22]	2015	Prospective case-control	55	2 (Astra Tech)	6, 12, 24 months	Ball, Locator attachments	Tissue response, patient satisfaction, retention
11.	Kappel et al. [23]	2015	Randomized clinical trial	78	2 (BEGO implants)	3, 6, 12, 24 months	Doldar bar, Locator	Tissue response, survival rate
12.	Cepa et al. [24]	2016	Randomized clinical trial	25	2 (Ankylos, Dentsply implants)	1, 2, 3 years	Ball, prefabricated conus	Survival rate, tissue response, patient satisfaction
13.	Albuquerque et al. [25]	2018	Randomized crossover clinical trial	24	2 (Straumann)	1 week, 3, 6, 12 months	Ball, Locator	Retention, patient satisfaction
14.	Dörsam et al. [26]	2021	Clinical pilot study	17	4 (Standard implants)	6, 12, 18, 24 months	Locator and CM LOC	Tissue response, patient satisfaction

Quality Assessment (Risk of Bias)

The Cochrane Collaboration tool (RevMan 5.4) was used to assess the risk of bias, categorizing studies as low, high, or unclear risk. Two studies had a high risk of bias in random sequence generation, while blinding participants and personnel showed an unclear risk across all studies.

Risk of Bias of the Included Studies

A detailed description of the risk of bias in the included studies has been shown in the "risk of bias" figures. Two studies (Cepa et al. [24] and Geckili et al. [22]) showed a high risk of bias in random sequence generation. The unclear risk was observed in the blinding of participants and personnel in all studies. The following studies are on random sequence generation: high risk of bias observed in two studies (Cepa et al. and Geckili et al.); low risk of bias observed in 11 studies (Albuquerque et al., Assad et al., Burns et al., Cristache et al., Gotfredsen and Holmet, Kappel et al., Kleis et al., Krennmair et al., Neart et al., and Wismeijer et al.); and unclear risk of bias observed in one study (Dörsam et al.). The following studies are on allocation concealment bias: low risk of bias observed in nine studies (Albuquerque et al., Assad et al., Geckili et al., Gotfredsen and Holmet, Dörsam et al., Kappel et al., Naert et al., and Wismeijer et al.) and unclear bias observed in five studies (Burns et al., Cepa et al., Cristache et al., Kappel et al., Kleis et al., and Krennmair et al.).

The blinding of participants and personnel revealed an unclear bias across all studies examined. The assessment of outcome blinding revealed a low risk of bias across all included studies.

For the incomplete outcome data, a low risk of bias was observed in 10 studies (Albuquerque et al., Assad et al., Cepa et al., Geckili et al., Gotfretsen et al., Dörsam et al., Kappel et al., Kleis et al., and Naert et al.). An unclear risk of bias was observed in four studies (Burns et al., Cristache et al., Krainnmair et al., and Wismeijer et al.). For the selective reporting bias, a low risk of bias was observed in 12 studies (Albuquerque et al., Assad et al., Cepa et al., Dörsam et al., Kappel et al., Kleis et al., Naert et al., Burns et al., Cristache et al., Krainnmair et al., and Wismeijer et al.). An unclear risk of bias was observed in Geckili et al. and Gotfredsen and Holmet.

Other Bias

**Figure 2:**
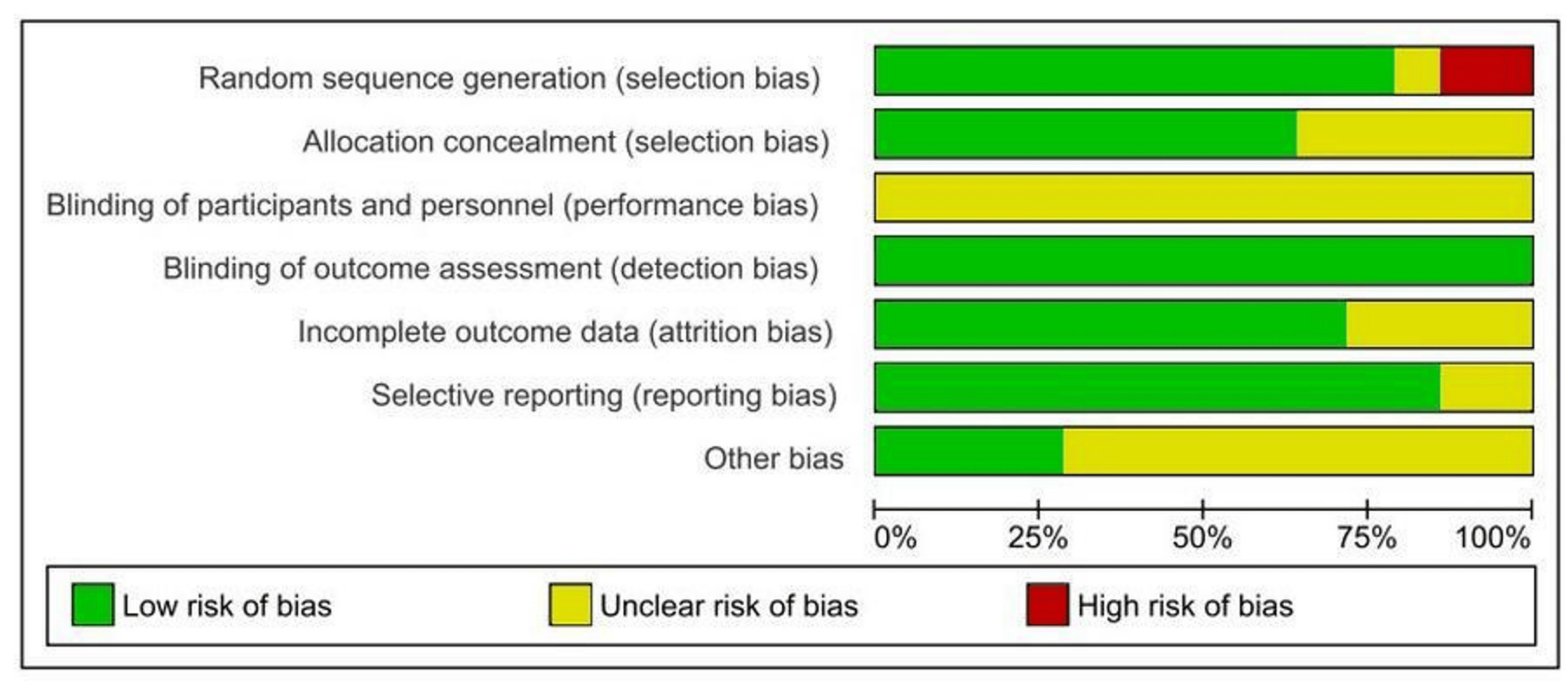
Risk of bias graph showing the judgments made by review authors about each risk of bias in the included studies, presented as percentages

**Figure 3:**
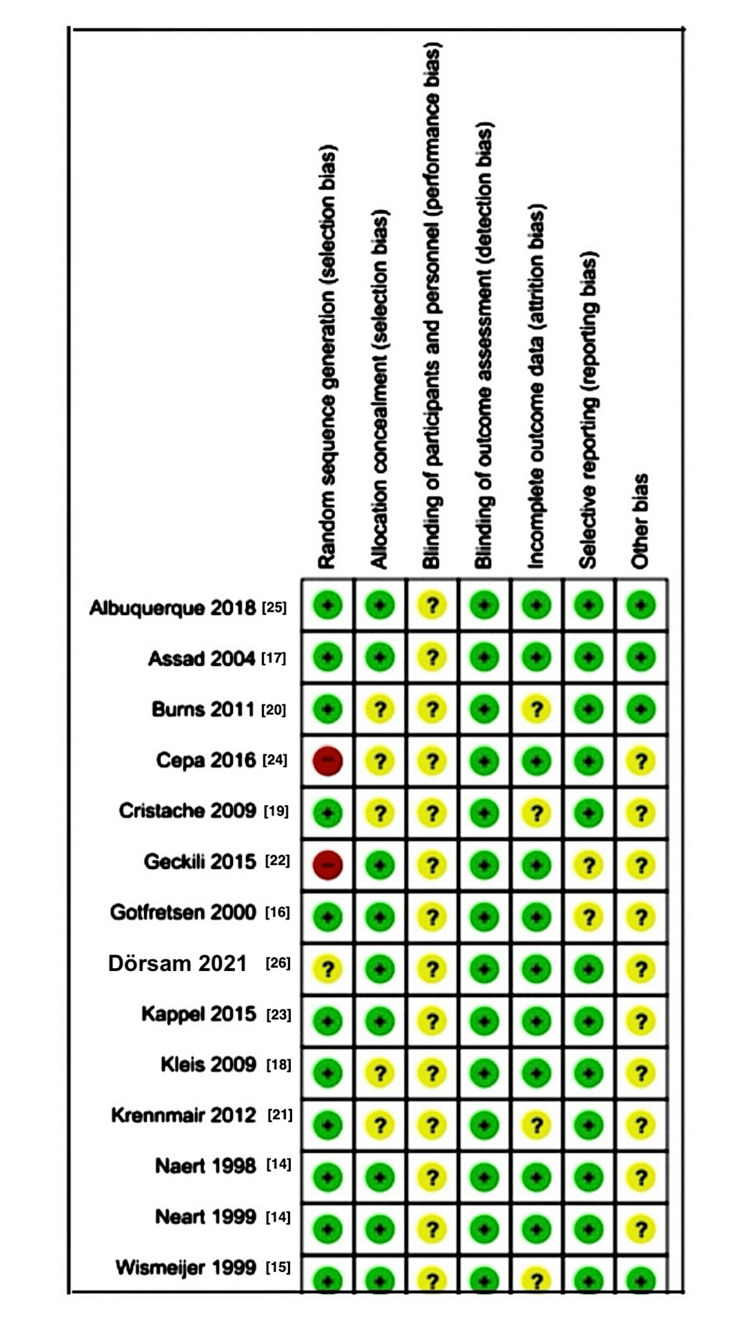
Risk of bias summary showing the judgments of review authors about the risk of bias for each included study

A low risk of bias was observed in four studies (Albuquerque et al., Assad et al., Burns et al., and Wismeijer et al.). An unclear risk of bias was observed in 10 studies (Cepa et al., Dörsam et al., Kappel et al., Kleis et al., Neart et al., Burns et al., Cristache et al., Krainnmair et al., Geckili et al., Gotfredsen and Holmet, and Dörsam et al.). Figures 2, 3 depict the risk of bias.

Meta-analysis

**Figure 4:**
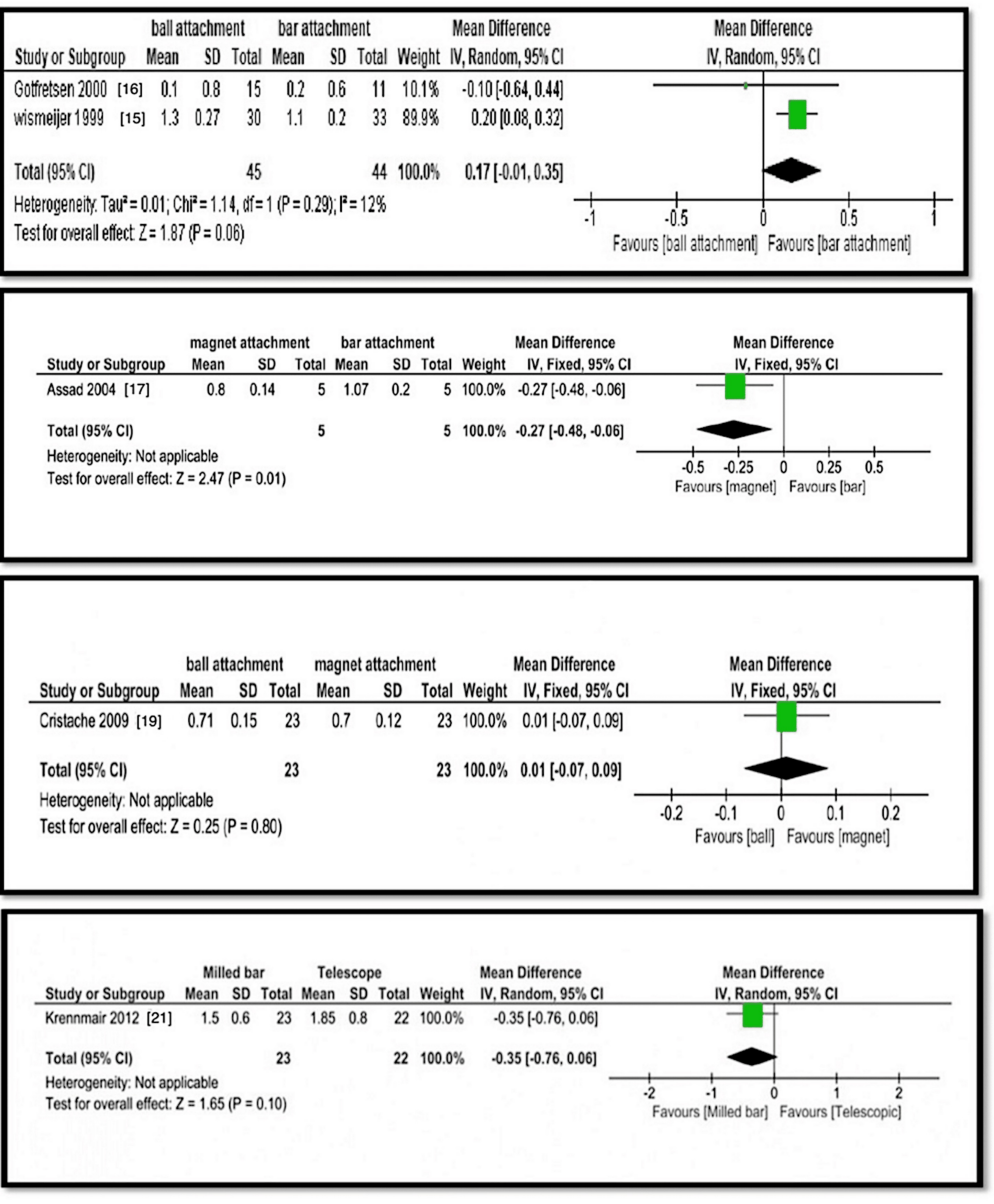
Analyses 1, 2, 3, and 4: comparison of ball and bar, magnet and bar, ball and magnet, and milled and telescopic attachments for bone loss around the implants

**Figure 5:**
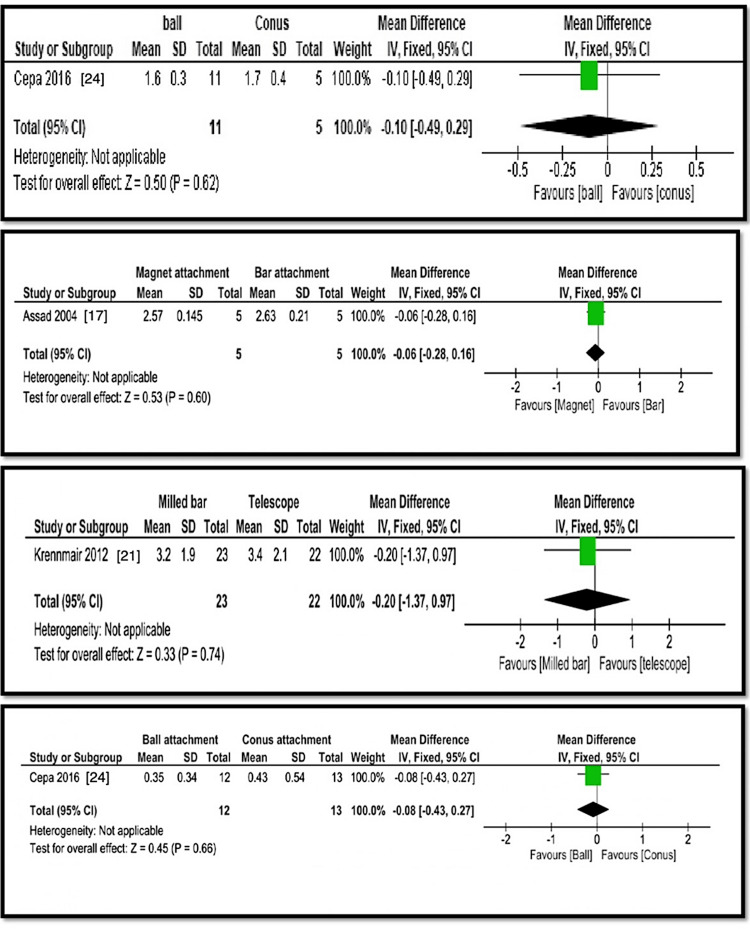
Analyses 5, 6, 7, and 8: comparison of ball and conus, magnet and bar, milled bar and telescope, and ball and conus attachments for bone loss around the implants

**Figure 6:**
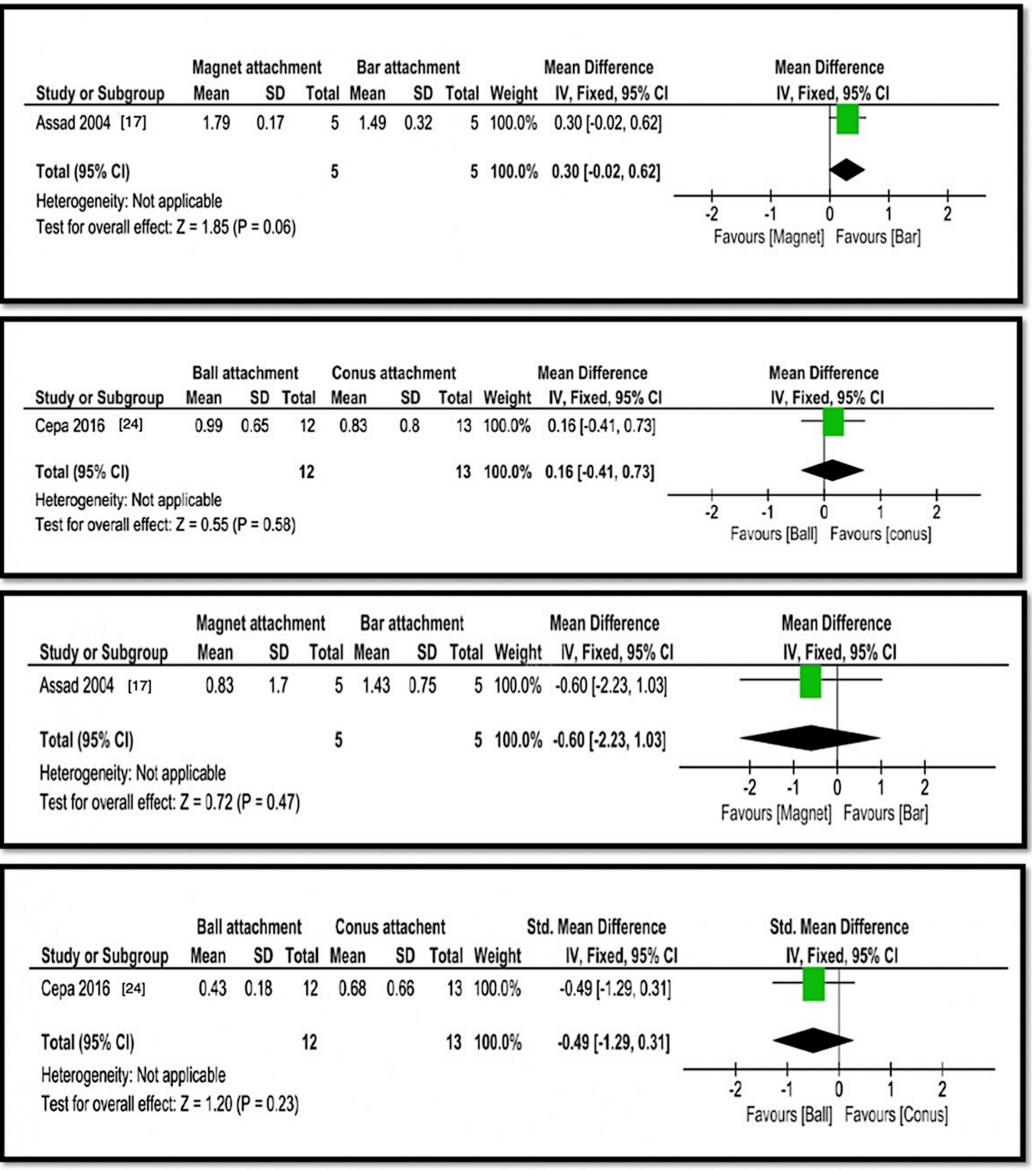
Analyses 9, 10, 11, and 12: comparison of magnet and bar, ball and conus, magnet and bar, and ball and conus attachments for bone loss around the implants

The analysis compared various implant overdenture attachment systems in terms of bone loss, probing depth, plaque index, and gingival index. Wismeijer et al. [15] and Gotfredsen et al. [16] found statistically significant differences in bone loss between ball and bar attachments, with ball attachments showing higher bone loss (1.1±0.8 mm and 1.3±0.27 mm) than bar attachments (0.2±0.6 mm and 1.1±0.2 mm) (p=0.002, Z=3.16). Assad et al. [17] found that magnet attachments had less bone loss (0.8±0.14 mm) than bar attachments (1.07±0.2 mm) (p=0.001, Z=2.47). Cristache et al. [19] reported no significant difference in bone loss between ball (0.71±0.15 mm) and magnet (0.7±0.12 mm) attachments (p=0.80, Z=0.25). Krennmair et al. [21] found no significant difference between milled bar (1.5±0.6 mm) and telescopic (1.85±0.8 mm) attachments (p=0.1, Z=1.65). Cepa et al. [24] found no significant difference in bone loss between ball (1.6±0.3 mm) and conus (1.7±0.4 mm) attachments (p=0.62, Z=0.5). Probing depth comparisons showed no significant differences between magnet (2.57±0.14 mm) and bar (2.63±0.21 mm) attachments (p=0.60, Z=53) or between milled bar (3.2±1.9 mm) and telescopic (3.2±2.1 mm) attachments (p=0.74, Z=0.33). Plaque index values were higher for magnet (1.79±0.17 mm) than bar (1.49±0.32 mm) attachments (p=0.06, Z=1.85), while no significant difference was found between ball (0.99±0.65 mm) and conus (0.83±0.8 mm) attachments (p=0.58, Z=0.55). Gingival index comparisons showed no significant differences between magnet (0.83±1.7 mm) and bar (1.43±0.75 mm) attachments (p=0.72, Z=47) or between ball (0.43±0.18 mm) and conus (0.68±0.66 mm) attachments (p=0.23, Z=1.20). Overall, bar attachments showed more bone loss, while magnet attachments had higher plaque index values, but statistical significance varied across comparisons (Figures 4-6). Table 2 presents a summary of evidence.

**Table 2:** Summary of evidence The table includes a concise explanation of the patient details and follow-up duration, the type of attachments used, the number of patients, tissue response, patient satisfaction, survival rate, and attachment retention. 2IBA: 2 implant ball attachments; 2ISB: 2 implant-supported single bars; 4ITB: 4 implants with triple bars; 2IBS: 2 implant-supported bar attachments; 2ISA: 2 implant-supported attachments; Dal-Ro: dual retaining orifice; TG-O: trans-gingival osseotite; 4IB: 4 implants supported by bar; 2IB: 2 implants supported by bar; CM LOC: Cendres+Métaux Locator

No.	Author name	Year	Follow-up	Type of attachment	No. of patients	Tissue response	Patient satisfaction	Survival rate	Retention
1	Naert et al. [14]	1999	5 years	Dolder bar, magnet, ball (Dala Bona)	Bar-12 Magnet-12 Ball-12	Ball> magnet> bar	Bar = ball> magnet	Bar> ball> magnet	Bar (1240) > ball (567) > magnet (110)
2	Wismeijer et al. [15]	1999	19 months	Ball (2IBA) Single bar (2ISB) Triple bar (4ITB)	2IBS-36, 2ISB-37, 4ITB-37	BI- 4ITB>2ISA>2IBA bone loss-4ITB > 2ISB = 2IBA	NR	NR	NR
3	Gotfredsen and Holm [16]	2000	5 years	Ball and bar attachment	Ball-15 Bar-11	Ball=bar	NR	100%	NR
4	Assad et al. [17]	2004	1.5 years	Magnet Bar	Magnet-5 Bar-5	Pocket depth bar (2.63) > magnet (2.57) Plaque index magnet (1.79) > bar (1.49) bleeding index bar (1.43) > magnet (0.83) bone loss bar > magnet	NR	NR	NR
5	Kleis et al. [18]	2009	-	Locator, Dal-Ro and TG-O	Locator-23 Dal-Ro-25 TG-O-8	Soft tissue recession Dal Ro>TG-O>Locator	Dal Ro=TG-O>Locator	NR	Dal Ro=TG-O>Locator
6	Cristache et al. [19]	2009	5 years	Ball, magnet	Ball-23 Magnet-23	Ball = magnet	NR	NR	NR
7	Burna et al. [20]	2011	1 year	4IB, 2IB, ball	4IB-10, 2IB-10, Ball-10	4IB>2IB>Ball	Ball > 4IB > 2IB	NR	4IB>Ball>2IB
8	Krennmair et al. [21]	2012	3 years	Milled bar, telescopic crown	Bar-20 Telescopic Crown-19	Bar = telescopic	NR	NR	NR
9	Geckili et al. [22]	2015	2 years	Ball and Locator	Ball-22 Locator-33	Ball = Locator	Ball = Locator	NR	Ball=Locator
10	Kappel et al. [23]	2015	2 years	Locator Dolder bar	Locator-23 Bar-23	Bone loss Locator > bar	NR	Bar-93.5% Locator-95.7%	Bar>Locator
11	Cepa et al. [24]	2015	3 years	Ball Conus	Ball-11 Conus-5	Plaque index ball (0.39±0.39)	Ball>Conus	100%	NR
12	Albuquerque et al. [25]	2018	1 year	Ball, Locator	Ball-12 Locator-12	NR	Ball> Locator	NR	Ball (20.2)> Locator(13.1)
13	Dörsam et al. [26]	2021	2 years	Locator CM LOC	Locator CM LOC	Locator=CMLOC	CM LOC> Locator	100%	NR

Discussion

Edentulism poses significant challenges to oral and general health, making prosthetic rehabilitation a crucial aspect of dental care [2]. Implant-supported overdentures enhance retention, stability, and patient satisfaction compared to conventional dentures, with attachment selection influenced by factors such as retention needs, cost, hygiene maintenance, inter-arch space, and bone availability [6,10]. Among the various attachment systems, bar attachments provide the highest retention but are more difficult to clean and require frequent maintenance due to clip fractures and adjustments. Ball attachments offer moderate retention, are easier to maintain, and have fewer soft tissue complications [11,12]. Magnet attachments, while resilient, exhibit the lowest retention and are associated with increased plaque accumulation and soft tissue inflammation, leading to lower patient satisfaction [11,27].

Locator and telescopic attachments strike a balance between retention and ease of maintenance, with telescopic attachments being particularly favored for their superior comfort, improved chewing efficiency, and reduced soft tissue complications [10]. Studies indicate that implant survival rates are largely independent of the attachment type, depending more on bone quality, implant placement, and maintenance [11]. However, complications such as attachment fractures, denture relining, and wear of retentive components vary across systems, with bar and ball attachments requiring more maintenance than locator or telescopic systems [10]. While retention forces differ among attachments, patient satisfaction remains consistently high with implant overdentures, with telescopic attachments ranking the highest due to their enhanced comfort, function, and long-term stability [10].​

## Conclusions

Implant-supported overdentures are a less invasive and more cost-effective option for mandibular edentulism than fixed prostheses. Ball attachments were the most commonly used, showing moderate tissue response, less than bar and magnet attachments, but slightly more than locator attachments. Bar attachments provided the highest retention but required >15 mm inter-arch space and had more soft tissue changes due to hygiene challenges. Magnet attachments had the lowest retention and highest soft tissue reactions, leading to the least patient satisfaction. Telescopic attachments had the highest patient satisfaction and effectively corrected implant angulation. Locator attachments were suitable for use in limited inter-arch space and for angulated implants with angles up to 50°, although they are less retentive compared to bar attachments. Splinted attachments improved load distribution and retention but were harder to clean, whereas non-splinted attachments had better tissue response. No significant association was found between implant survival rates and attachment systems.
